# Quantitative Proteomics Reveals Antibiotics Resistance Function of Outer Membrane Proteins in *Aeromonas hydrophila*

**DOI:** 10.3389/fcimb.2018.00390

**Published:** 2018-11-06

**Authors:** Zujie Yao, Lina Sun, Yuqian Wang, Ling Lin, Zhuang Guo, Dong Li, Wenxiong Lin, Xiangmin Lin

**Affiliations:** ^1^Fujian Provincial Key Laboratory of Agroecological Processing and Safety Monitoring, College of Life Sciences, Fujian Agriculture and Forestry University, Fuzhou, China; ^2^Key Laboratory of Crop Ecology and Molecular Physiology Fujian Agriculture and Forestry University, Fujian Province University, Fuzhou, China; ^3^Shanghai Key Laboratory of Plant Functional Genomics and Resources, Shanghai Chenshan Plant Science Research Center, Chinese Academy of Sciences, Shanghai Chenshan Botanical Garden, Shanghai, China; ^4^Institute of Life Sciences, Jiangsu University, Zhenjiang, China

**Keywords:** oxytetracycline resistance, sarcosine-insoluble proteins, outer membrane protein, *Aeromonas hydrophila*, quantitative proteomics

## Abstract

Outer membrane proteins (OMPs) play essential roles in antibiotic resistance, particularly in Gram-negative bacteria; however, they still have many unidentified functions regarding their behavior in response to antibiotic stress. In the current work, quantitative tandem mass tag labeling-based mass spectrometry was used to compare the outer membrane related proteins between an oxytetracycline-resistant (OXY-R) and its original control stain (OXY-O) in *Aeromonas hydrophila*. Consequently, a total of 261 commonly altered proteins in two biological repeats were identified including 29 proteins that increased and 28 that decreased. Gene ontology analysis showed that the expression of transport proteins was significantly reduced, and translation-related proteins were downregulated in the OXY-R strain. After using western blotting to validate selected altered proteins, eight OMP-related genes were knocked out and their roles in antibiotic resistance were further evaluated. The survival assays showed that some mutants such as Δ*AHA_4281*, Δ*AHA_2766*, Δ*AHA_2282*, Δ*AHA_1181*, and Δ*AHA_1280* affected the susceptibility of *A. hydrophila* to antimicrobials. Moreover, the minimum inhibitory concentration assay showed that these candidate mutants also respond differently to other types of antibiotics. Our results reveal several novel outer membrane related proteins of *A. hydrophila* that play important roles in antibiotic resistance, and as such, may be helpful for screening studies to identify novel drug targets.

## Introduction

In Gram-negative bacteria, the asymmetrical outer membrane (OM) is unique structure and plays a pivotal role in bacterial survival (Wu et al., 2013; Rollauer et al., 2015). The outer membrane, as a natural barrier of Gram-negative bacteria, endows the bacteria with more resistance to harsh surroundings, such as extreme acidity, alkalinity, and temperature, and various toxicities (Nikaido, 2003; Dong et al., 2014; Pagès et al., 2015; Srinivasan et al., 2015). The outer membrane proteins (OMPs), including the transmembrane proteins and lipoproteins, are the major components of the OM, and the phospholipids of inner leaflet and lipopolysaccharide (LPS) of outer leaflet also simultaneously constitute this membrane (Dong et al., 2014; Liao et al., 2017). In addition, many proteins that are predicted to be located on the inner membrane, periplasm, extracellular space, and even the cytoplasm have also been frequently identified in the OM fraction in many studies (Veith et al., 2014; Ottman et al., 2016). This may be caused technological contamination due to current protein extraction technologies, and the OM fractions are comprised of other proteins for another important reason: these proteins do the binding on OM or make a complex with outer membrane/protein for another important reason (Cao et al., 2012; Ottman et al., 2016). Nevertheless, these outer membrane related proteins play important roles in protecting the complex cellular environment from agents that damage the peptidoglycan wall including antibiotics (Guo et al., 2014; Srinivasan et al., 2015).

The antibiotic-resistant properties of OMPs can essentially be grouped into two fundamental functions. One is reducing OM permeability to prevent the uptake of antibiotics (most of which are hydrophilic or amphiphilic) across the OM via the outer membrane porins (Masi and Pagès, 2013). For instance, two homologous OMPs in *Escherichia coli*, OmpC and OmpF, can control the uptake of various antibiotics. Loss of OmpF leads to significantly increased resistance to β-lactam drugs such as ampicillin and cefoxitin, whereas the absence of OmpC reduces the minimum inhibitory concentration (MIC) of carbapenem and cefoxitin antibiotics (Mortimer and Piddock, 1993; Moya-Torres et al., 2014; Tran et al., 2014). The Resistance Nodulation Cell Division (RND) superfamily of Gram-negative efflux pump is involved in the exportation of biological and microbial metabolites; such well-studied tripartite RND systems are found, for example in *Pseudomonas aeruginosa* (MexAB-OprM) and in *E.coli* (AcrAB-TolC) (Nikaido, 2009; Daury et al., 2016).

In our previous study, we used traditional two-dimensional gel electrophoresis-based proteomics methods to identify several novel OMPs, such as LamB, OmpT, and OmpA, which are involved in antibiotic resistance in *E. coli*. Particularly with regard to LamB, which facilitates the uptake of maltose, the deletion of this gene in *E. coli* increased the MIC of multiple drugs, suggesting that it is a nonspecific channel for antibiotics (Lin et al., 2014). However, given the fact that there are many types of OMPs in Gram-negative bacteria, which have multiple biological functions such as nutrient transport and stress responses, the antibiotic-resistant functions of these proteins remain largely unclear.

*Aeromonas hydrophila* is a Gram-negative fish pathogen that is present in a variety of aquatic environments and causes huge economic losses in aquaculture (Li et al., 2016a). It was recently reported that this pathogen also infects humans and other animals such as amphibians and reptiles, and can even lead to death (Hoel et al., 2017; Song et al., 2017; AlYahya et al., 2018). The increasingly severe situation of the emergence of *A. hydrophila* strains with high resistance to antibiotics has aroused public attention (Done et al., 2015; Watts et al., 2017). Understanding the mechanisms underlying antibiotic resistance in this bacterium would be helpful for the development of novel drugs (Hernould et al., 2008). In this study, we evaluated the antibiotic-resistant properties of OM proteins in this pathogen. Outer membrane related proteins containing the integral OMPs were extracted from an oxytetracycline-resistant (OXY-R) and its control strain (OXY-O) of *A. hydrophila* using the sarcosine-insoluble method. Then the differential expression of proteins was compared using the tandem mass tag (TMT) labeling-based quantitative proteomics method combined with high-resolution mass spectrometry (Tran et al., 2014). Western blotting was used to confirm changes in the expression of selected OMPs, which were initially identified in our proteomics result. Evaluation of bacterial survival among related gene mutants indicated their biological functions in antibiotic resistance. The results of this study provide novel insights into the role of OMPs in the antibiotic resistance mechanisms of *A. hydrophila*, and may be helpful for screening studies to identify novel drug targets.

## Materials and methods

### Bacterial strains, plasmids, and cultivation

In this study, *A. hydrophila* ATCC7966, *E. coli* MC1061, *E. coli* S17-1, and pRE112 plasmid were stored in our laboratory. An OXY-R strain (*A.h*-OXY-R) was induced from original (control) *A. hydrophila* ATCC7966 (*A.h*-OXY-O) as previously described (Liu et al., 2015). All bacteria were separately cultured in lysogeny broth (LB, yeast extract 5 g/L, tryptone 10 g/L, and sodium chloride 10 g/L, pH 7.2) medium at 30°C, with the exception of *E. coli*, which were cultured at 37°C.

### Sarcosine-insoluble protein extraction

The OMPs were prepared using a previously described sarcosine-insoluble method (Peng et al., 2017). Briefly, each colony of *A. hydrophila* and the OXY-R strain were incubated in 5 mL LB medium overnight, and then diluted in 100 mL fresh LB medium at a ratio of 1:100 and subsequently cultured until the optical density at 600 nm (OD_600_) reached 1.0. The cultures were harvested via centrifuging for 20 min at 10,000 x g, 4°C, and then the bacterial cells were washed with cold phosphate buffered saline (PBS, pH 7.4) for three times. The cell pellets were resuspended in the 10 mL ultrasonic buffer (50 mM Tris-HCl, pH 7.4, 1 mM PMSF) and disrupted with intermittent sonic oscillation for a total of 30 min at 9 s intervals on ice. Subsequently, the cell debris and unbroken cells were separated by centrifugation at 8,000 x g for 20 min at 4°C. Then the supernatant was centrifuged at 100,000 x g for 1 h at 4°C in the Optima LE-80 K Ultracentrifuge (Beckman, Palo Alto, CA, USA). After the pellet was dissolved with 2% sodium lauroyl sarcosine (in 50 mM Tris-HCl, pH 7.5) for 40 min at room temperature (RT), the pellets were ultracentrifuged again at 100,000 x g for 1 h at 4°C. Finally, the precipitate was dissolved in an appropriate volume of SDT buffer (4 % SDS, 0.1 M DTT [dithiothreitol], and 0.5 M triethylammonium bicarbonate buffer [TEAB, pH 8.5]). The protein concentration was determined using the Bradford method and then stored at −20°C until subsequent use.

### In-solution digestion and TMT labeling

The proteins were digested with trypsin after being reduced with DTT and alkylated with iodoacetamide using a filter-aided sample preparation method (Tanca et al., 2013; Li et al., 2016b). After washing three times with 0.5 M TEAB followed by fractionation using the 10 kDa ultrafiltration system (Millipore, Billerica, MA, USA), about 100 μg digested peptide from each group, including two biological replicates, was taken out for further labeled using sixplex TMT isobaric and isotopic mass-tagging kits (Thermo Fisher Scientific, MA, USA), which was performed according to instructions from the kit (https://www.thermofisher.com/order/catalog/product/90101?SID=srch-srp-90101).

### Proteomics analysis by liquid chromatography–tandem MS

Labeled peptides were re-suspended in 0.1 % formic acid and submitted to analysis with the AB/Sciex TripleTOF 5600 Plus Mass Spectrometer (AB SCIEX, Concord, ON, Canada) combined with the NanoAcquity ultraperformance liquid chromatography (UPLC) system (Waters Inc., Milford, MA, USA), which were performed using the same parameters as previously described (Li et al., 2016b). Briefly, the labeled peptides were firstly trapped with reverse-phase Symmetry C18 trapping column (180 μm × 20 mm, Waters) and subsequently switched into the analytical 1.7 μm BEH130 C18 (100 μm × 100 mm, Waters). The digested peptides were separated beyond 90 min at a flow rate of 300 nL/min utilizing the 8–25% gradient of solvent B containing 0.1% formic acid in acetonitrile and then eluted onto the mass spectrometer. The data was acquired in the positive-ion mode coupled with an ion-spray voltage of 2.3 kV, curtain gas of 30, an interface heated temperature of 150°C. Regarding to the information dependent acquisition (IDA), survey scans in the mass range of 350 to 1,500 m/z were collected in 100 ms. And, when the abundance threshold of 200 counts per second (counts/s), the 30 product ion scans with charge state of 2–5 were selected. Dynamic exclusion of precursor ions was set as 1/2 of the peak width (22 s).

The raw data were analyzed by Mascot Server 2.4 (Matrix Science, Framingham MA, USA) combined with Scaffold 4.3.4 (Proteome Software, Portland, OR, USA) against the *A. hydrophila* ATCC7966 database in Uniprot. Each experiment was done in two biological replicates and two technical replicates. The .mgf files were converted from the .wiff and.scan files utilizing Ms_data_converter_V1.3 (AB SCIEX) and the data of two technical replicates were merged together and searched on a Mascot searching engine. The search parameters were set as previously described (Li et al., 2016b) and included cysteine carbamidomethylation and TMT labeling as fixed modification, methionine oxidation as variable modification. The maximum missed cleavage allowance for trypsin digestion was two, then peptide tolerance and MS/MS tolerance were respectively set to ± 0.05 Da and ±0.03 Da. And, the mass tolerances of monoisotopic precursor and the level of fragment ion were set as 50 ppm and 0.1 Da, respectively. We selected proteins with at least two unique peptides that matched as reliable candidates for further quantitation. Proteins with an average isobaric tag for relative and absolute quantification ratio of *A.h*-OXY-R to *A.h*-OXY-O that was higher than 1.5 or lower than 0.667, and with a false discovery rate < 1 % in both biological replicates, were considered significantly changed. The mass spectrometry proteomics data have been deposited to the ProteomeXchange Consortium via the PRIDE (Vizcaino et al., 2016) partner repository with the dataset identifier PXD009622 and 10.6019/PXD009622.

### Bioinformatics analysis

We used the online Venn tool to display the overlap of identified proteins from two biological replicates (Khan and Mathelier, 2017). The subcellular location prediction of commonly altered proteins were predicted by Cell-PLoc 2.0 package of the online Gneg-multi software (http://www.csbio.sjtu.edu.cn/bioinf/Cell-PLoc-2/) with the default setting (Chou and Shen, 2006). Gene Ontology (GO) and Kyoto Encyclopedia of Genes and Genomes analyses of pathways corresponding to the altered proteins were performed using OmicsBean online software (Li et al., 2017). We also combined STRING v.10 and Cytoscape v.3.3 software to predict and visualize the protein-protein interactions (PPIs) of differential proteins according to the website instructions (Lopes et al., 2010; Szklarczyk et al., 2015).

### Purification of recombinant proteins

The selected four genes were cloned into the pET-32a plasmid. Then, these recombinant proteins were overexpressed in *E. coli* BL21 (DE3) and purified with Ni-NTA column affinity chromatography according to previous study (Peng et al., 2016). In brief, the overexpressed strains were cultured overnight in 5 mL of LB medium containing 100 μg/mL ampicillin, and the bacterial suspensions were 1% (v/v) diluted to incubate in 200 mL fresh LB medium until the OD_600_ reached 0.6. The fusion proteins were induced to express for 7 h at 20°C using 1 mM isopropy-β-D-thiogalactoside (IPTG). The cell cultures were centrifuged to harvest at 10,000 x g, 4°C for 10 min, and rinsed with PBS (pH 7.4) for three times. Resolving in binding buffer (25 mM Na_2_HPO_4_•12H_2_O, 10 mM NaH_2_PO_4_•2H_2_O, 500 mM NaCl, 5 mM imidazole), the cell pellets were broken up with intermittent sonication for 30 min on ice bath at 9 s intervals. After separating the cell debris by centrifugation at 10,000 x g for 30 min at 4°C, the fusion protein supernatants were loaded on the Ni-NTA resin (Ni Sepharose 6 Fast Flow, GE Healthcare, Uppsala, Sweden) columns. Unbound proteins were washed off from columns with 10 mL binding buffer for five times. These columns containing fusion proteins were rinsed once with one volume of elution buffer I of 50 mM imidazole, and eventually eluted with 1 mL elution buffer II of 500 mM imidazole twice. The protein concentrations were determined by BCA Assay Kit (Thermo Fisher Scientific) and then submitted to Hua An Biotechnology Co., Ltd (HuaBio, Hangzhou, China) to produce specific rabbit antisera.

### Western blotting to validate the proteomics analysis

Total bacterial proteins were separated by sodium dodecyl sulfate-polyacrylamide gel electrophoresis (SDS-PAGE) and were electrophoretically transferred to polyvinylidene fluoride (PVDF) membranes in transfer buffer (Bio-Rad, 1X Tris/Glycine Buffer) at RT as previously described (Zhang et al., 2017). Briefly, PVDF membranes were blocked in 5% (w/v) nonfat milk in phosphate-buffed saline with 0.1 % (v/v) Tween 20 (PBST) for 2 h at RT prior to incubation with polyclonal rabbit antibodies (1:1,000 dilution) against the target protein at 4°C for 12 h. After washing with PBST, the membranes were incubated for 1 h with horseradish peroxidase-conjugated goat anti-rabbit antibody (ComWin, Beijing, China) (1:5,000 dilution). The immunostained proteins were visualized using Clarity™ Western ECL Substrate (Bio-Rad, Hercules, CA, USA), and images were acquired with the ChemiDoc MP imaging system with Image Lab software (Bio-Rad). Coomassie R-350 was used to stain the PVDF membranes after blotting as a loading control.

### Construction of deletion mutants

We constructed genetic deletion mutants using the recombination and pRE112 suicide vector system as previously described (Yu et al., 2005). First, about 500 base pairs (bp) of upstream and downstream flanking sequences of the target gene open reading frame were amplified and fused into the plasmid pRE112 by overlapping PCR. Then the construct was firstly introduced into *E. coli* MC1061 in order to raise the transformation efficiency and quickly propagate, and then transformed into *E. coli* strain S17-1 for conjugal transfer. Next the plasmid was introduced into *A. hydrophila* by bacterial conjugation with S17-1. Single recombinants were selected on LB agar with ampicillin (100 μg/mL) and chloramphenicol (30 μg/mL) for the first homologous recombination. Subsequently, transformants were spread onto LB agar with 20% (w/v) sucrose accompanied by the second homologous recombination. Finally, the positive colonies were verified by PCR and sequencing. The primer sequences of constructing the knockout mutants were provided in Table S2.

### Assay of bacterial survival

Knockout and wild-type bacterial strains were grown in 5 mL LB medium overnight, and then diluted into fresh LB medium containing different OXY concentrations (0, 5, 10, 20, and 40 μg/mL) at a ratio of 1:100, respectively. The bacterial growth curves of related strains were measured at intervals of 1 h at OD_600_ by Bioscreen-C (Oy Growth Curves AB Ltd., Helsinki, Finland). In every group of treatment with OXY, the survival ratio of each mutant was estimated the statistical significance comparing with the wild- type strain based on one-way ANOVA. The *p* value < 0.05 meant significant difference and exhibited with the asterisk (^*^).

### MIC assay

The MICs of *A. hydrophila* and related mutants were measured by the doubling dilution method as previously described with modifications (Peng et al., 2017). Briefly, 200 μL doubled diluted antibiotics in fresh LB medium were added to each well of the 96-well microtiter polystyrene tray with and without antibiotics (as a negative control) and 10 μL containing about 1 × 10^5^ bacteria in LB were added to each well. All treatments were repeated at least three times followed by incubation at 30°C for 24 h.

## Results

### Identification and characterization of sarcosine-insoluble proteins in *A. hydrophila*

**Figure 1:**
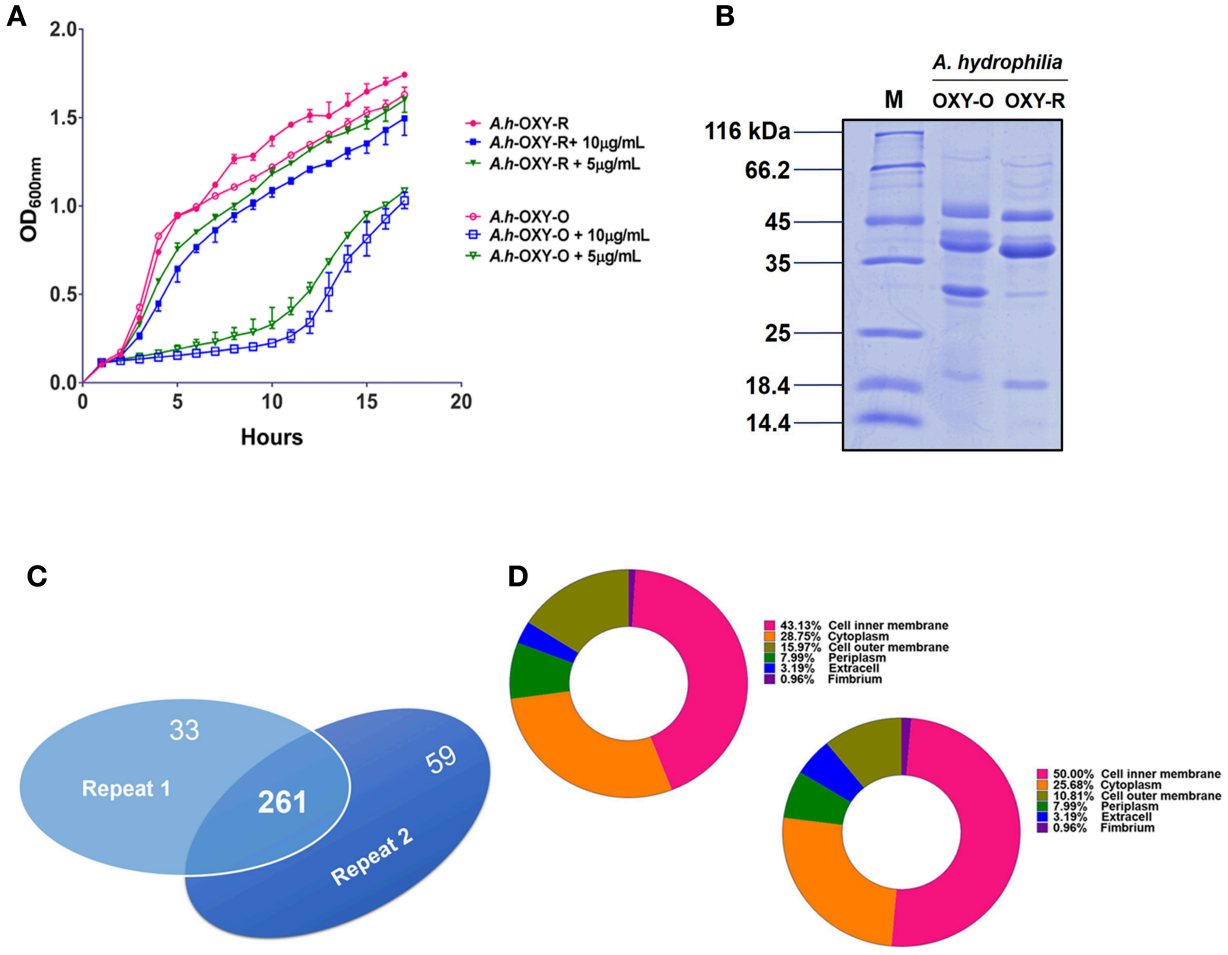
Comparative analysis of OM fractions from *A. hydrophila* ATCC 7966 under OXY stress. **(A)** Growth curve of *A.h*-OXY-R and *A.h*-OXY-O at concentrations of 0, 5,10 μg/mL; **(B)** Coomassie Blue-stained SDS-PAGE of OMPs in *A.h*-OXY-R and *A.h*-OXY-O, Lane M contains molecular mass standards; **(C)** Venn diagram showing overlap of the identified proteins by LC-MS/MS between two biological repeats in this study; **(D)** Prediction of subcellular localization of identified (left panel) and altered proteins (right panel) by online Gneg-multi-software.

The antibiotic resistance characteristics of the induced *A. hydrophila* strain (*A.h*-OXY-R) were compared to its original control stain. As shown in Figure 1A, compared to OXY- susceptible strain (*A.h*-OXY-O), the OXY-resistant strain (*A.h*-OXY-R) showed high survival under 5 and 10 μg/mL OXY treatment, which indicated that *A.h*-OXY-R had acquired OXY resistance. To investigate the role of *A.h*-OXY-R OMPs in antibiotic resistance, sarcosine-insoluble proteins were extracted and separated by SDS-PAGE. Significant differences in bands between *A.h*-OXY-R and *A.h*-OXY-O were observed, suggesting that these proteins play an important role in antibiotic resistance (Figure 1B). Furthermore, two comparison samples were in-solution digested, and differentially expressed proteins were quantified with TMT labeling-based quantitative proteomics combined with high-resolution MS. Result showed that the 320 and 294 proteins were, respectively identified by TMT labeling in two biological replicates (repeat 1 and repeat 2, respectively) and a total of 261 proteins overlapped between both biological replicates (Figure 1C and Table S1). And, some significantly differential OMPs of *A.h*-OXY-R were presented in Table 1. Moreover, the correlations of ratio of identified and altered proteins between them were analyzed and displayed in Figure S1. Results showed a moderate correlation between the log2 ratios of the common quantitative data from two biological replicates with the regression coefficients >0.485 (Figure S1A). The correlation of the log2 ratios of commonly significantly altered proteins between both groups was moderately higher (ρ = 0.915, Figure S1B), which indicates the MS stability is considerable. We predicted the subcellular location of these 261 proteins using Gneg-mPLoc online software. The results showed that there were about 71.3% cell membrane proteins, including inner membrane proteins (43.1%), OMPs (16%), periplasmic proteins (8%), extracellular proteins (3.2%), and fimbrial proteins (0.96%), in the sarcosine-insoluble fractions (Figure 1D, left panel). Beside these, a total of 57 altered proteins were identified in these overlapped proteins including 7 altered OMPs (Figure 1D, right panel).

**Table 1:** Significantly differentially expressed OMPs in *A. hydrophila* ATCC 7966 OXY-resistant strain by LC MS/MS.

**Accession number**	**Gene name**	**Description**	**Match peptides**	**Coverage (%)**	**Repeat 1 ratio**	**Repeat 2 ratio**
A0KHH1_AERHH	*AHA_1181*	Outer membrane protein assembly factor BamA	33	48.6	0.532	0.516
A0KLQ6_AERHH	*AHA_2699*	Agglutination protein	32	69.5	0.470	0.142
A0KNY2_AERHH	*AHA_3509*	Outer membrane efflux protein	5	14.4	0.539	0.410
A0KFM8_AERHH	*AHA_0521*	Outer membrane usher protein	12	18.8	0.613	0.255
A0KHS0_AERHH	*AHA_1280*	Major outer membrane protein OmpAII	16	55.0	0.218	0.185
A0KMJ4_AERHH	*AHA_2991*	Outer membrane efflux protein	10	42.0	0.266	0.298
A0KLX3_AERHH	*AHA_2766*	Outer membrane protein	18	36.7	0.378	0.156

### Functional classification annotation and PPI network prediction of altered proteins

**Figure 2:**
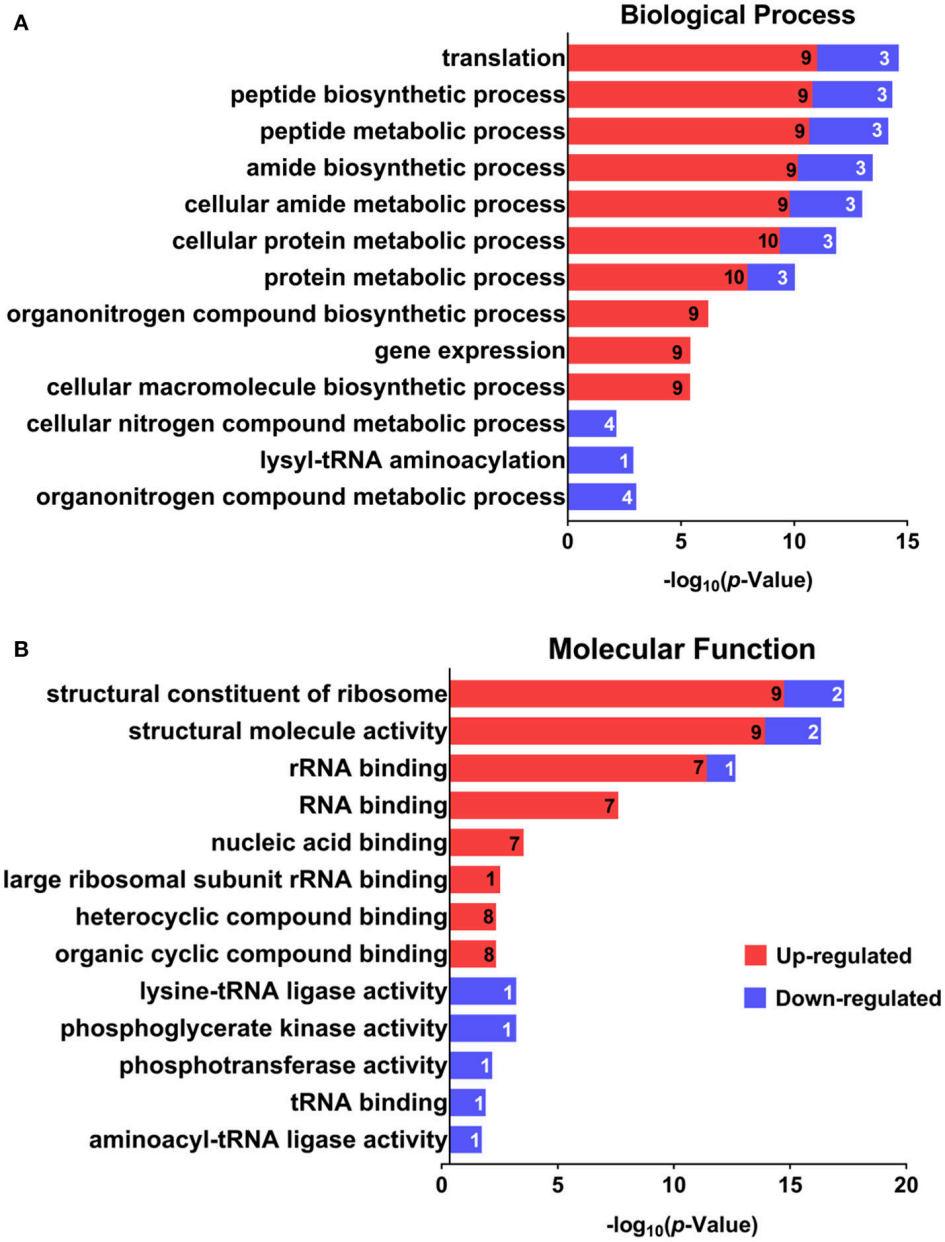
GO analysis of differentially expressed proteins. GO analysis of differentially expressed proteins in *A. hydrophila* and *A. hydrophila-R*. Red color bars indicate upregulated proteins and blue color bars indicate downregulated proteins related to biological processes **(A)** and molecular functions **(B)**. Explanatory information on the functional enrichment and numbers of involved proteins are all listed on the left and right, respectively, behind the bars. Terms of the same category are grouped by *P*-values and shown on the y-axis (log_10_ scale on the y-axis).

**Figure 3:**
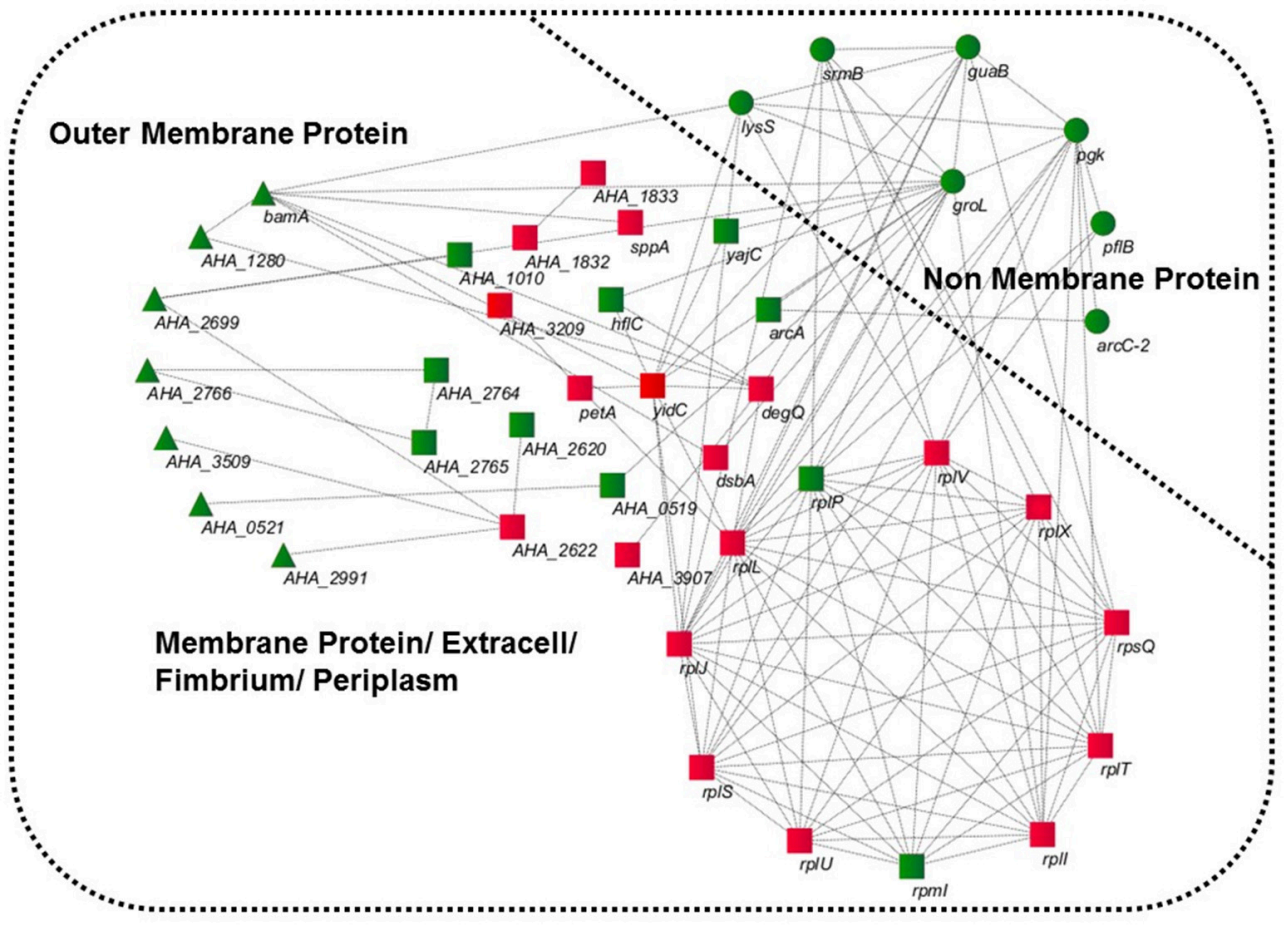
STRING software prediction of PPI networks. Enrichment analysis of altered proteins in 16MIC OXY, Circles (green) represents non-membrane proteins including SrmB, GuaB, Pgk, ArcC-2, PlfB, GroL, LysS; triangles (green) represent outer membrane proteins including BamA (*AHA_1181*), A0KLQ6 (*AHA_2699*), A0KNY2 (*AHA_3509*), A0KFM8 (*AHA_0521*), A0KMJ4 (*AHA_2991*), A0KLX3 (*AHA_2766*), and OmpAII (*AHA_1280*); squares represent inner membrane proteins or periplasmic proteins, mainly including RplP, RpmI, RplI, RplT, RpsQ, RplX, RplJ, RplS, RplL, RplU, RplV, DegQ, and YidC. All of gene names are in black, and the red and green in three shapes are the overexpressed and downregulated in proteomics, respectively.

GO was performed to determine the functional classification of the altered proteins between *A.h*-OXY-R and *A.h*-OXY-O strains. In the biological process category, upregulated proteins were mostly related to translation-related processes such as translation, peptide/protein biosynthesis, and metabolism. Only proteins related to biological processes such as organonitrogen compound biosynthesis, gene expression, and cellular macromolecule metabolism were upregulated, whereas only proteins related to the metabolism of cellular nitrogen/organonitrogen and lysyl-tRNA aminoacylation were downregulated (Figure 2A). With regard to classification of molecular function, proteins related to translation-related processes such as ribosomes, rRNA binding, and nucleic acid binding were largely enriched as well (Figure 2B). It is generally considered that PPI networks play important roles in the mechanisms underlying bacterial resistance. We used STRING software to further research the PPI network. As shown in Figure 3, the PPI network included at least seven OMPs including the outer membrane assembly factor protein BamA (*AHA_1181*), agglutination protein A0KLQ6 (*AHA_2699*), outer membrane efflux protein A0KNY2 (*AHA_3509*), outer membrane usher protein A0KFM8 (*AHA_0521*), major OMP OmpAII (*AHA_1280*), outer membrane efflux protein A0KMJ4 (*AHA_2991*), and outer membrane protein A0KLX3 (*AHA_2766*), all of which were significantly decreased in the OXY-R strain. Meanwhile, the downregulation of seven cytoplasmic proteins, namely ATP-dependent RNA helicase (SrmB), inosine-5'-monophosphate dehydrogenase (GuaB), lysine-tRNA ligase (LysS), GroEL protein (GroL), phosphoglycerate kinase (Pgk), outer-membrane protein A (ArcC-2), and formate acetyltransferase (PlfB) were also observed. Moreover, 11 ribosomal subunit proteins were altered including RplP, RplV, RplX, RplT, RpsQ, RplI, RpmI, RplU, RplS, RplJ, and RplL most of which were upregulated with the exception of RpmI and RpIP. It is worth noting that some proteins annotated as cytoplasmic or inner membrane proteins were found in the OM fraction as well, such as ribosomal proteins and GroEL protein, which might be related to the isolating method of OMPs (Thein et al., 2010).

### Western blotting validation of selected proteins identified in proteomics results

**Figure 4:**
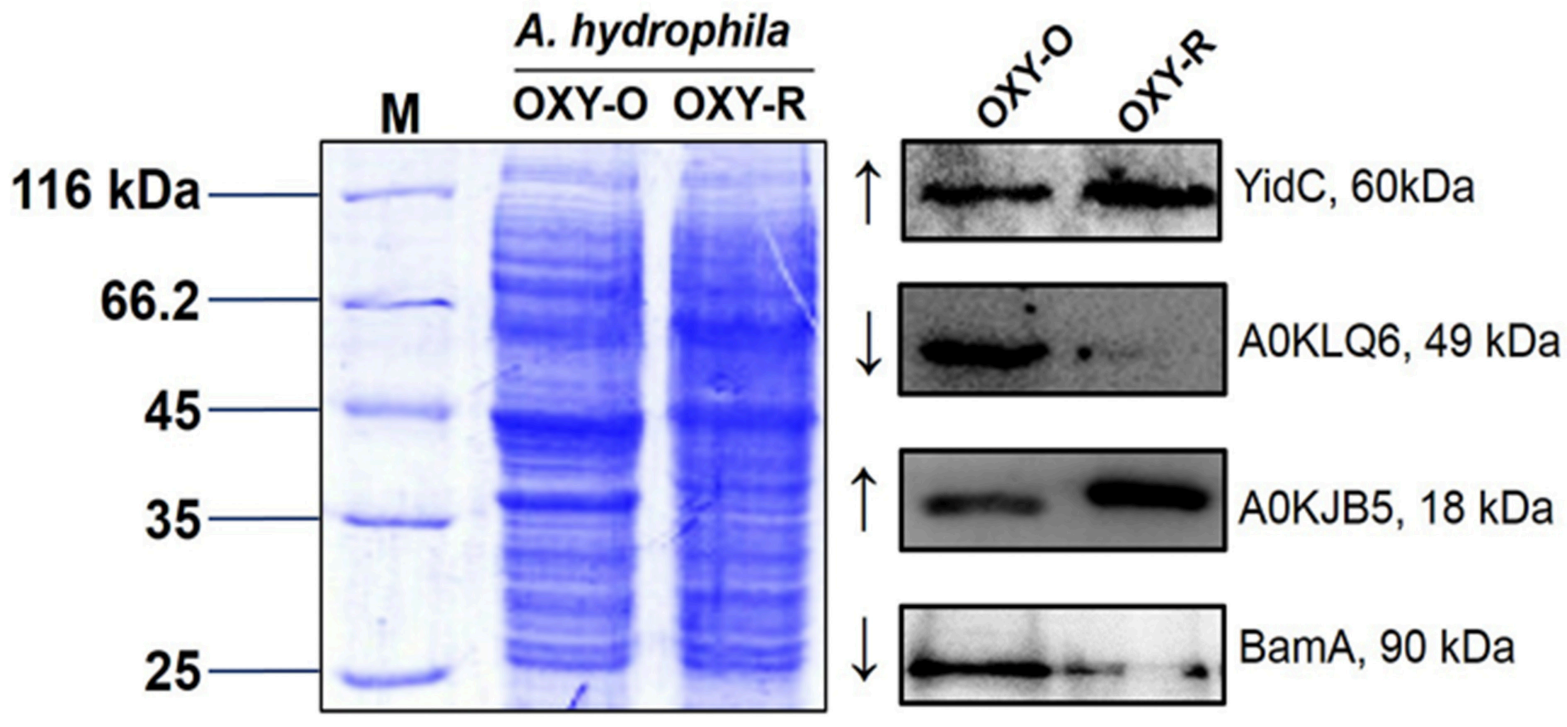
Western blotting to confirm proteomics analyses. Western blot analysis for YidC, A0KLQ6, A0KJB5, and BamA expression compared to OXY-O and OXY-R. Coomassie staining was used as the loading control (on the left). The expected size of YidC, A0KLQ6, A0KJB5, and BamA was 60, 49, 18, and 90 kDa, respectively.

To validate the proteomics data, three altered sarcosine-insoluble proteins were selected including membrane protein insertase (YidC), agglutination protein (A0KLQ6), uncharacterized protein (A0KJB5) and outer membrane protein assembly factor BamA for confirmation by western blotting (Figures S7, S8). The results showed that A0KLQ6 and BamA were downregulated whereas YidC and A0KJB5 were clearly upregulated. Thus the western blotting results were consistent with the MS results shown in Figure 4 and Figure S6.

### OM related proteins affect antimicrobial capabilities upon OXY stress

**Figure 5:**
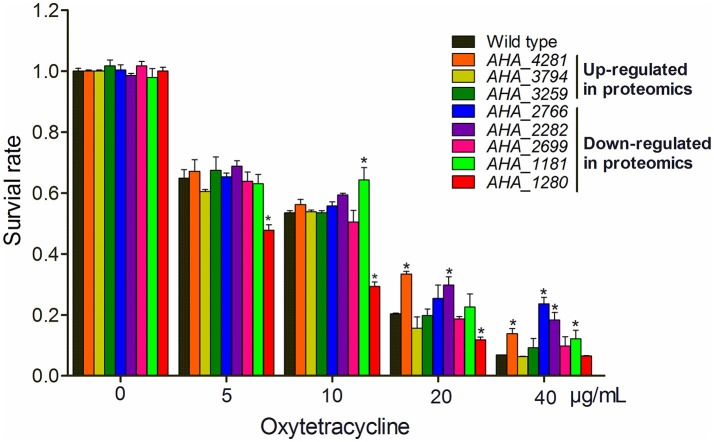
Histograms displaying survival capabilities of selected mutants upon OXY stress. The survival capabilities of eight mutants, including Δ*AHA_1181*, Δ*AHA_2282*, Δ*AHA_3794*, Δ*AHA_4281*, Δ*AHA_1280*, Δ*AHA_3259*, Δ*AHA_2699*, and Δ*AHA_2766* were calculated under OXY treatment of different concentrations (0, 5, 10, 20, and 40 μg/mL). ^*^P < 0.05.

To gain a better understanding of the role of OM related protein in antibiotic resistance, eight genes encoding membrane protein insertase (*AHA_4281, yidC*), uncharacterized protein (*AHA_3794*), penicillin-binding protein (*AHA_3259*), OMP (*AHA_2766*), uncharacterized protein (*AHA_2282*), agglutination protein (*AHA_2699*), OMP assembly factor BamA (*AHA_1181*), and major OMP OmpAII (*AHA_1280*) were knocked out and their antibiotic resistance abilities were evaluated by the antimicrobial survival capability assay. As shown in Figure 5, Δ*AHA_4281 (yidC)*, Δ*AHA_2766*, Δ*AHA_2282*, and Δ*AHA_1181* showed a higher growth ratio with high doses of OXY than the wild-type strain, Δ*AHA_1181* expression was sharply decreased in the presence of serial OXY concentrations, and Δ*AHA_3794*, Δ*AHA_3259*, and Δ*AHA_2699* did not show any significant changes upon antibiotic stress. Moreover, we further observed the survival capabilities of *A.h*- OXY-O and Δ*AHA_1181* treated with a series of OXY concentrations (0, 5, 10, 20, and 40 μg/mL) for 12 h by colony counting. The following antibiotics resistance function validation from *bamA* knocked out strain also confirmed that BamA did play important role on antibiotics resistance (Figure 5 and Figure S5). Simultaneously, the growth curves of these mutant strains under serial concentrations of OXY stress were measured for 12 h (Figure S4). Consequently, in addition to higher concentration treatment, the Δ*AHA_4281*, Δ*AHA_2282*, and Δ*AHA_2766* increased the antibiotic resistance to OXY comparing with the wild-type strain (*A.h*-OXY-O), whereas Δ*AHA_1280* showed the tendency to improve the susceptibility to OXY. Nevertheless, Δ*AHA_3794* had similar resistance to wild type with OXY, and Δ*AHA_2766* and Δ*AHA_3259* displayed no significant difference in MICs to OXY. The fluctuant survival rates of different mutants indicate the different roles of these proteins in antibiotic resistance. In addition, the several verified results about knockout mutants used in the present work were presented in Supplementary Materials, including the validation by colony PCR (Figure S2), comparisons from sequencing results (Figure S3) and their base sequences (File S1). These validations indicated that these eight genes were successfully deleted in *A. hydrophila*, respectively.

### OM related proteins in *A. hydrophila* affect the MICs of different types of antibiotics

**Figure 6:**
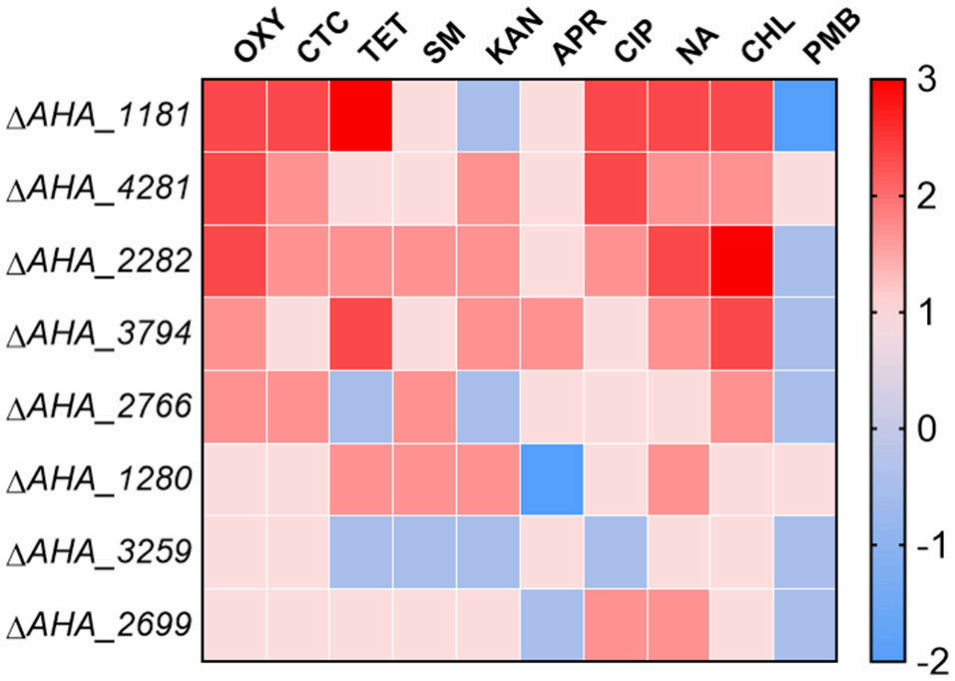
Heatmap displays MICs of *A. hydrophila* ATCC7966 and gene deletion strains. MICs of eight gene deletion mutants under the treatment of different antibiotics, including oxytetracycline (OXY), chlortetracycline (CTC), tetracycline (TET), streptomycin (SM), kanamycin (KAN), apramycin (APR), ciprofloxacin (CIP), nalidixic acid (NA), chloramphenicol (CHL), and polymyxin B sulfate (PMB). The fold changes of MICs are shown on the right side. Color grading represents a serial of change folds of different antibiotics.

To further validate whether these eight OM related proteins affect the antimicrobial capabilities of different types of antibiotics, we measured the MICs of the related mutants against ten types of antibiotics including oxytetracycline (OXY), chlortetracycline (CTC), tetracycline (TET), streptomycin (SM), kanamycin (KAN), apramycin (APR), ciprofloxacin (CIP), nalidixic acid (NA), chloramphenicol (CHL), and polymyxin B sulfate (PMB). As shown in Figure 6, the MICs of Δ*AHA_1181* to tetracycline and quinolone antibiotics had significant increase, and this mutant simultaneously showed high sensitivity to KAN and PMB, which suggested that BamA may have an important impact on these antibiotic resistances. Similarly, the MICs of Δ*AHA_4281* to OXY and CIP were also elevated, suggesting that this protein plays an important role in both kinds of antibiotic resistances as well. Meanwhile, we found that Δ*AHA_2282* had distinctly higher MICs to OXY, CHL, and NA, indicating that it had more antibiotic-resistant properties. Furthermore, Δ*AHA_3794* has displayed similar sensitivity or resistance to Δ*AHA_2282* with tetracycline antibiotics and CHL. Moreover, we also observed behaviors of other mutants with different antibiotics, and the results indicated that these mutants had no significant difference in MICs to OXY, including Δ*AHA_1280*, Δ*AHA_2699*, Δ*AHA_2766*, and Δ*AHA_3259*. In general, the results showed that *A. hydrophila* and knockout strains had different trends and produced distinct degrees of resistance and sensitivity to different antibiotics, which may be associated with some of their specific physiological and physical properties.

## Discussion

In the past several decades, the phenomenon of antibiotic resistance has grown more severe with multiple affects including those to human life and the environment. *A. hydrophila* infections are specifically associated with large-scale fish farming, where the development of resistance is a large concern (Li et al., 2016b; Yao et al., 2016). It is well known that bacterial OMPs play important roles in antibiotic resistance, but few reports have focused on their behaviors in *A. hydrophila*. Thus, in this research study, we compared differentially expressed sarcosine-insoluble proteins between OXY-R and OXY-susceptible strains using TMT labeling-based proteomics. Of the 261 identified proteins, 57 were altered (29 increased and 28 decreased). GO analysis showed that the upregulated proteins were associated with biological processes such as translation, and peptide or protein synthesis, in accordance with the molecular mechanisms underlying OXY inhibition of the translation process and accumulation of translation materials such as ribosome subunits (Chukwudi, 2016). Meanwhile, proteins involved in cellular macromolecular metabolism and lysyl-tRNA aminoacylation were downregulated, indicating that intracellular metabolism is involved in the mechanisms of antibiotic resistance as previously described (Peng et al., 2015; Chaliotis et al., 2017; Zampieri et al., 2017). Moreover, western blotting of selected altered proteins confirmed our OM proteomics results.

To better understand the antibiotic-resistant roles of altered proteins in our OM proteome, we constructed eight gene deletion mutants that were altered in the OXY-R strain including four integral OMPs (gene names: *AHA_1280, AHA_2699, AHA_4281*, and *AHA_2766*) and four membrane proteins (*AHA_1181, AHA_3259, AHA_3794*, and *AHA_2282*), and then tested their antibiotic susceptibilities. All of the altered integral OMPs were significantly decreased in *A.h*-OXY-R including the major OMP OmpAII (*AHA_1280*). This is a highly conserved major β-barrel porin in many bacterial species that has multiple functions such as participation in adhesion and invasion, functions as an immune target, and functions as a receptor for colicin and bacteriophage (Liao et al., 2017). Several studies have shown that OmpA is closely linked with antibiotic resistance; for example, OmpA deletion causes more resistance to carbapenem antibiotics in *Acinetobacter baumannii* (Zarrilli et al., 2013). In this study, the decrease of OmpAII in proteomics suggests that it may reduce OM permeability or affect the influx of antibiotics as an antibiotics channel to obtain antibiotic resistance. The *ompAII* mutant caused decreased growth upon OXY stress, but showed no significant change in MIC. Meanwhile, the MICs of the *ompAII* mutant to other antibiotics in this study showed no difference as well, indicating that OmpAII in *A. hydrophila* only slightly contributes to antibiotic resistance.

Another protein that was significantly decreased in OXY-R strains was the outer membrane assembly factor BamA (*AHA_1181*), which is involved in the β-barrel assembly machinery for the recognition of OMP folding and assembly, as well as maintenance of the bacterial cell envelope. In addition, the BamA complex functions as a facilitator of OMP folding and allows the cell envelope to act as a negative regulator of the response to various antimicrobial agents (Ricci et al., 2013; Zarrilli et al., 2013). When *bamA* is knocked out in *E. coli*, the unfolded β-barrel protein cannot be correctly inserted into the outer membrane and result in bacterial death (Doerrler and Raetz, 2005; Werner and Misra, 2005; Malinverni et al., 2006). Interestingly, the growth curves seen in Figure S4 indicated the *bamA* mutant in *A. hydrophila* displayed the severe growth defect that it could still be alive but is far from happy in normal conditions; nevertheless, *A. hydrophila* lacking *bamA* could reduce the influx of some kinds of antibiotics, such as those used in this study, through the OMP channel to obtain antibiotic resistance.

Proteomics analysis showed the sharply decreasing expression of agglutination protein (*AHA_2699*), which was validated by western blotting. Homology analysis indicates that this protein is homologous to the TolC channel protein, which is a typical outer membrane efflux protein in *E. coli* and most likely has a similar function in *A. hydrophila* (Krishnamoorthy et al., 2013). Surprisingly, this agglutination protein had decreased expression in the OXY-R strain, but its deletion mutant displayed no significant differences in MICs to OXY, KAN, and CHL compared with the wild type. However, the MIC of Δ*AHA_2699* to CIP was 8-fold higher than the control, probably because it functions as a specific channel for CIP pass through. In addition, it may also be related to biofilm formation and adherence as previously reported in agglutination protein AggA and a similar protein, LapE, that is involved in agglutination and adherence in *P. putida* and *P. fluorescens* (Buell and Anderson, 1992; Hinsa et al., 2003).

In this study, the OMP (*AHA_2766*) which belongs to the MtrB/PioB family was decreased in *A.h*-OXY-R and the corresponding deletion mutant displayed resistance to OXY and CHL, sensitivity to KAN, and no difference to CIP. The homology analysis suggested that this protein might be the outer membrane porin composed of 28 transmembrane beta strands, and may also have the largest number of beta strands among all known outer membrane porins (Jiao and Newman, 2007). However, the antibiotic resistance role of this protein needs further investigation.

In addition to these integral OMPs, four membrane proteins were altered in the OM proteome of the OXY-R strain, namely YidC (*AHA_4281*, upregulated), penicillin-binding protein (*AHA_3259*, downregulated), and two uncharacterized proteins (*AHA_3794* and *AHA_2282*, both downregulated). YidC is involved in membrane protein insertion in bacteria including the biogenesis of penicillin-binding protein (Price et al., 2010; de Sousa Borges et al., 2015). Its upregulation may impact the composition of proteins located at the membrane, eventually affecting susceptibility to antibiotics. Interestingly, although the penicillin-binding protein is reportedly involved in penicillin resistance (Li et al., 2016) and was decreased in *A.h*.-OXY-R in this study, we did not found any alterations in MIC or survival capability, suggesting that its role in antibiotic resistance may be weak. We also evaluated the antibiotic-resistant function of two uncharacterized proteins. Although *AHA_3794* showed no difference in growth rate in the bacterial survival capability assay, both proteins displayed higher MICs to other antibiotics, especially TET and CHL, suggesting a novel function for both unknown proteins in bacterial antibiotic resistance.

## Conclusion

The results of this current study demonstrated the effects of OXY resistance on sarcosine-insoluble proteins in the *A. hydrophila* strain using TMT-labeling quantitative proteomics. Biological processes such as translation and transportation were found to play very important roles in antibiotic stress. Differential expression level of selected OMPs were verified by western blot analysis. In addition, mutants of selected proteins were used to assess survival capability and MIC assays. We found several novel OMPs involved in antibiotic resistance. Thus, this study furthers our understanding of the functions of OM-related proteins in OXY resistance.

## Author contributions

All the authors contributed extensively to the work presented in this manuscript. ZY and LS contributed equally to this work. XL and WL designed the experiments. ZY, LS, and YW generated experimental data and wrote the manuscript. LL, ZG, DL, and XL conceived the work and critically review the manuscript. The authors declare no competing financial interest.

### Conflict of interest statement

The authors declare that the research was conducted in the absence of any commercial or financial relationships that could be construed as a potential conflict of interest.

## Supplementary material

Supplementary Table S1 Identification and quantitative outer membrane proteomics results of *A.h*-OXY-O and *A.h*-OXY-R strains using TMT labeling proteomics analysis (XLS).

Supplementary Table S2 Sequences of the primer pairs used in this study for constructing the genetic deletion mutants.

Figure S1 Correlation analysis of overlapped proteins and differential proteins (log2 ratio) between two biological replicates. **(A,B)** the correlation of commonly quantitative and altered proteins from two biological replicates, respectively.

Figure S2 Validation of the knockout mutants by colony PCR amiplified with the primer pairs P7 and P8. The sizes of eight genes are 2424 bp (*AHA_1181*), 1002 bp (*AHA_1280*), 1638 bp (*AHA_2282*), 1344 bp (*AHA_2699*), 1992 bp (*AHA_2766*), 1191 bp (*AHA_3259*), 570 bp (*AHA_3794*), and 1647 bp (*AHA_4281*), respectively. Lanes M1 and M2, the DL 5,000 and DL 2,000 DNA marker; lanes 3, 7, 8, 10, 14, 16, 18, and 20, the PCR products of wild-type strains amplified with the primers P7 and P8 as a positive control, and their expected sizes of fragments were 2966 bp (lane 3), 3133 bp (lane 7), 2847 bp (lane 8), 3594 bp (lane 10), 1810 bp (lane 14), 2396 bp (lane 16), 2166 bp (lane 18), and 3165 bp (lane 20); lanes 1, 2, 4, 5, 6, 9, 11, 12, 13, 15, 17, and 19, the possible colonies of knockout mutants pick out for colony PCR verification with primers P7 and P8; in addition to lanes 4-6 (1789 bp), the expected sizes of remaining ones were 1328 bp (lane 1-2), 1200 bp (lane 9), 1170 bp (lane 11), 1240 bp (lane 12-13), 1205 bp (lane 15), 1164 bp (lane 17), and 1173 bp (lane 19), which means the right results that these genes have been deleted in *A. hydrophila*.

Figure S3 Comparisons from sequencing results of the genetic deletion mutants by BLAST online software. The possible mutants were sequenced with P7 and P8 primers and compared to their genes in *A. hydrophila* used on online software BLAST.

Figure S4 Growth curves of the mutants and *A.h*- OXY-O under serial concentrations of OXY stress. The growth curves of wile-type strain and mutants were measured for 12 h in different concentrations of 0, 5, 10, 20, and 40 μg/mL OXY.

Figure S5 Survival capabilities of *A.h*- OXY-O and Δ*AHA_1181* treated with a series of OXY concentrations (0, 5, 10, 20, and 40 μg/mL) for 12 h and observed by colony counting. **(A)** the CFU of them under each concentration of OXY treatment (log10 scale on the y-axis). **(B)** the survival ratio of wild type and Δ*AHA_1181* mutant when treated with serial dilutions of OXY concentrations for 12 h.

Figure S6 Western blotting analysis for differential outer membrane proteins contained the prestained protein ladder. The prestrained protein marker was used to detect the molecular weights of proteins, and the expected size of BamA. YidC, A0KLQ6, and A0KJB5 was 90 kDa, 60 kDa, 49 kDa, and 18 kDa, respectively.

Figure S7 The validation of the specificity of antibodies by Western blotting. Western blotting was used to detect the specificity of these polyclonal antibodies in *A.h*-OXY-O. Coomassie staining was used as the loading control (on the right). The expected size of A0KLQ6, A0KJB5, YidC, and BamA was 49 kDa, 18 kDa, 60 kDa, and 90 kDa, respectively.

Figure S8 The specificity of anti-YidC and BamA was further validated in knock-out strains by western blotting. The specificity of two antibodies were examined in Δ*AHA_4281 (yidC)* and Δ*AHA_1181 (bamA)*, and coomassie staining was used as the loading control.

File S1 The base sequences of knockout mutants analyzed by the primer pairs P7 and P8. Each mutant was sequenced with P7 and P8 primer, and their base sequences were presented in text.
