# Supplementary material for: Quantitative Proteomics Reveals Antibiotics Resistance Function of Outer Membrane Proteins in Aeromonas hydrophila
Source: Front Cell Infect Microbiol. 2018 Nov 6;8:390. doi: 10.3389/fcimb.2018.00390 (PMC6232253; doi:10.3389/fcimb.2018.00390)
Supplement: Supplementary Table S2 — Sequences of the primer pairs used in this study for constructing the genetic deletion mutants. [file Table_2.DOCX]

**Table S2. Primers for constructing the genetic deletion mutants**

| **Primer** | **Sequence (5’→ 3’)** | **Description** |
| --- | --- | --- |
| *AHA_1181*-P1 | cgatcccaagcttcttctagaCCAGCTGGTCGGTTTCGTC | Forward primer of upstream sequence |
| *AHA_1181*-P2 | cagagtccgtccTTTGTTCTTATGTGTCCTTGTTGATG | Reverse primer of upstream sequence |
| *AHA_1181*-P3 | agaacaaaGGACGGACTCTGCCCCCA | Forward primer of downstream sequence |
| *AHA_1181*-P4 | catgaattcccgggagagctcCGGACTGACGGCGACCGT | Reverse primer downstream sequence |
| *AHA_1181*-P5 | ATGGCTGTTAAAAAAGCATTGGT | Forward primer of *AHA_1181* gene sequence |
| *AHA_1181*-P6 | TTAGAAGGTACGGCCGATGTTG | Reverse primer of *AHA_1181* gene sequence |
| *AHA_1181*-P7 | GTCCACCAGGTGCAGCAGTC | Locating at the front of the upstream sequence |
| *AHA_1181*-P8 | AGCACCAGATCGTAACCATTGCTC | Locating at the back of the downstream sequence |
| *AHA_1280*-P1 | catgaattcccgggagagctcATCTCCTGCCTGGCTCCG | Forward primer of upstream sequence |
| *AHA_1280*-P2 | ccaaggtgacCGTTTTATCCTCGTTGAGTTTGC | Reverse primer of upstream sequence |
| *AHA_1280*-P3 | ggataaaacgGTCACCTTGGTGGCCGAAA | Forward primer of downstream sequence |
| *AHA_1280*-P4 | cgatcccaagcttcttctagaCAATCACCGACAGGGTGGC | Reverse primer downstream sequence |
| *AHA_1280*-P5 | ATGAAAATGAAAATGGCACC | Forward primer of *AHA_1280* gene sequence |
| *AHA_1280*-P6 | TTACTGGGTGACTTGCTGTAC | Reverse primer of *AHA_1280* gene sequence |
| *AHA_1280*-P7 | TTGCCAACTTCCTGGTCAGCAAAG | Locating at the front of the upstream sequence |
| *AHA_1280*-P8 | ACTTGACGTGGTAGACATAACCGC | Locating at the back of the downstream sequence |
| *AHA_2282*-P1 | catgaattcccgggagagctcTTGCTGGAAGAGCTGCTGGC | Forward primer of upstream sequence |
| *AHA_2282*-P2 | CTACTTCCTTAGTCCTGATGTTTCAACG | Reverse primer of upstream sequence |
| *AHA_2282*-P3 | catcaggactaaggaagtagAAAGTTGTGTCACTAAAAATTAATGCG | Forward primer of downstream sequence |
| *AHA_2282*-P4 | cgatcccaagcttcttctagaAAGATCACCTTCAAGCGCGTC | Reverse primer downstream sequence |
| *AHA_2282*-P5 | ATGAAAAAGCAATTATCCATGCTGGC | Forward primer of *AHA_2282* gene sequence |
| *AHA_2282*-P6 | TTAGCGCTGGAAACGGCGCAGC | Reverse primer of *AHA_2282* gene sequence |
| *AHA_2282*-P7 | ACTTCCTGTTTGAACCAAAACGGGC | Locating at the front of the upstream sequence |
| *AHA_2282*-P8 | TGGTCGGTTTCTTCCTTGGCTGGA | Locating at the back of the downstream sequence |
| *AHA_2699*-P1 | catgaattcccgggagagctcCCGCTCAAGGGCACCATC | Forward primer of upstream sequence |
| *AHA_2699*-P2 | atgcgtactccaGGTATCTCCAGTCATGTTGTTTTTAGA | Reverse primer of upstream sequence |
| *AHA_2699*-P3 | gagataccTGGAGTACGCATCAGGTTTGATG | Forward primer of downstream sequence |
| *AHA_2699*-P4 | cgatcccaagcttcttctagaCCAATAGTCTTTTTTGCGCCA | Reverse primer downstream sequence |
| *AHA_2699*-P5 | ATGAAGAAGACGATTGTTGCC | Forward primer of *AHA_2699* gene sequence |
| *AHA_2699*-P6 | TTACTGTTTAGCTTGCCAATCGGCC | Reverse primer of *AHA_2699* gene sequence |
| *AHA_2699*-P7 | GGCTCGAGAAAAATCAGTTCAGG | Locating at the front of the upstream sequence |
| *AHA_2699*-P8 | TGTCTGTAGAGCGTCATAGGTTAATCA | Locating at the back of the downstream sequence |
| *AHA_2766*-P1 | catgaattcccgggagagctcTGTACCAGCTGCCACAGCCT | Forward primer of upstream sequence |
| *AHA_2766*-P2 | aatcgtgcctGACTTCTCTCCTCAGCGCCG | Reverse primer of upstream sequence |
| *AHA_2766*-P3 | gagagaagtcAGGCACGATTGCACACCG | Forward primer of downstream sequence |
| *AHA_2766*-P4 | cgatcccaagcttcttctagaATGTACCAGCACATGATGGTGAA | Reverse primer downstream sequence |
| *AHA_2766*-P5 | ATGAAATACTCAACACTTTGGCTCTC | Forward primer of *AHA_2766* gene sequence |
| *AHA_2766*-P6 | TTAGAACTTGTAGAGCCAGGAGACG | Reverse primer of *AHA_2766* gene sequence |
| *AHA_2766*-P7 | TGGAGAAGCAGAACAGCGTCTGTC | Locating at the front of the upstream sequence |
| *AHA_2766*-P8 | TCTACCAGGAGTCGAGCCAGCTG | Locating at the back of the downstream sequence |
| *AHA_3259*-P1 | catgaattcccgggagagctcTCGTCAAACCCGCCAAACC | Forward primer of upstream sequence |
| *AHA_3259*-P2 | ttgtgcgcgggCTTGAAATTCCTGGAGCCTGG | Reverse primer of upstream sequence |
| *AHA_3259*-P3 | aatttcaagCCCGCGCACAAACCTGAC | Forward primer of downstream sequence |
| *AHA_3259*-P4 | cgatcccaagcttcttctagaTATGTTCCTTGCTCTGGGCG | Reverse primer downstream sequence |
| *AHA_3259*-P5 | ATGTCCAAATTTGCCCGTTC | Forward primer of *AHA_3259* gene sequence |
| *AHA_3259*-P6 | TTACTTGAACCAGCTGGAGACC | Reverse primer of *AHA_3259* gene sequence |
| *AHA_3259*-P7 | CCATGAAGCCCATCGAGATGACG | Locating at the front of the upstream sequence |
| *AHA_3259*-P8 | CTGGCAAATAATCAGTCGCCCCG | Locating at the back of the downstream sequence |
| *AHA_3794*-P1 | catgaattcccgggagagctcAGCGACGCTCAAGACCTCC | Forward primer of upstream sequence |
| *AHA_3794*-P2 | tttCATTGACCTCGCTTGGGCT | Reverse primer of upstream sequence |
| *AHA_3794*-P3 | cccaagcgaggtcaatgAAAATGGAACGGGAGGCCT | Forward primer of downstream sequence |
| *AHA_3794*-P4 | cgatcccaagcttcttctagaAACTCGACAACGGCCTGCT | Reverse primer downstream sequence |
| *AHA_3794*-P5 | ATGAAGAAACAGATGATGGCGCTGG | Forward primer of *AHA_3794* gene sequence |
| *AHA_3794*-P6 | TCAGGCGCCCTTGCGCACCTCTGC | Reverse primer of *AHA_3794* gene sequence |
| *AHA_3794*-P7 | TTATGCCAATGCGGAGAAGGAGCA | Locating at the front of the upstream sequence |
| *AHA_3794*-P8 | CGTCTCGAATACCAGTACACCACC | Locating at the back of the downstream sequence |
| *AHA_4281*-P1 | catgaattcccgggagagctcCCGGGCCGCTTCTCCGCA | Forward primer of upstream sequence |
| *AHA_4281*-P2 | gggagcgaAGTCTTCAGTTTCTTCGCTTTGG | Reverse primer of upstream sequence |
| *AHA_4281*-P3 | aaactgaagactTCGCTCCCGATGGCAAGA | Forward primer of downstream sequence |
| *AHA_4281*-P4 | cgatcccaagcttcttctagAGGTCGGCGATGGCCTCGG | Reverse primer downstream sequence |
| *AHA_4281*-P5 | ATGGAATCACAACGCAATCTCATCC | Forward primer of *AHA_4281* gene sequence |
| *AHA_4281*-P6 | TTAGCTGCGGGTATGCAGGCCTTTC | Reverse primer of *AHA_4281* gene sequence |
| *AHA_4281*-P7 | CGTGCCCGCCTGGTAGTCTAA | Locating at the front of the upstream sequence |
| *AHA_4281*-P8 | CGGGTGAGACTCTCCACCAGC | Locating at the back of the downstream sequence |
