# Supplementary figures and images for: Quantitative Proteomics Reveals Antibiotics Resistance Function of Outer Membrane Proteins in Aeromonas hydrophila
Source: Front Cell Infect Microbiol. 2018 Nov 6;8:390. doi: 10.3389/fcimb.2018.00390 (PMC6232253; doi:10.3389/fcimb.2018.00390)

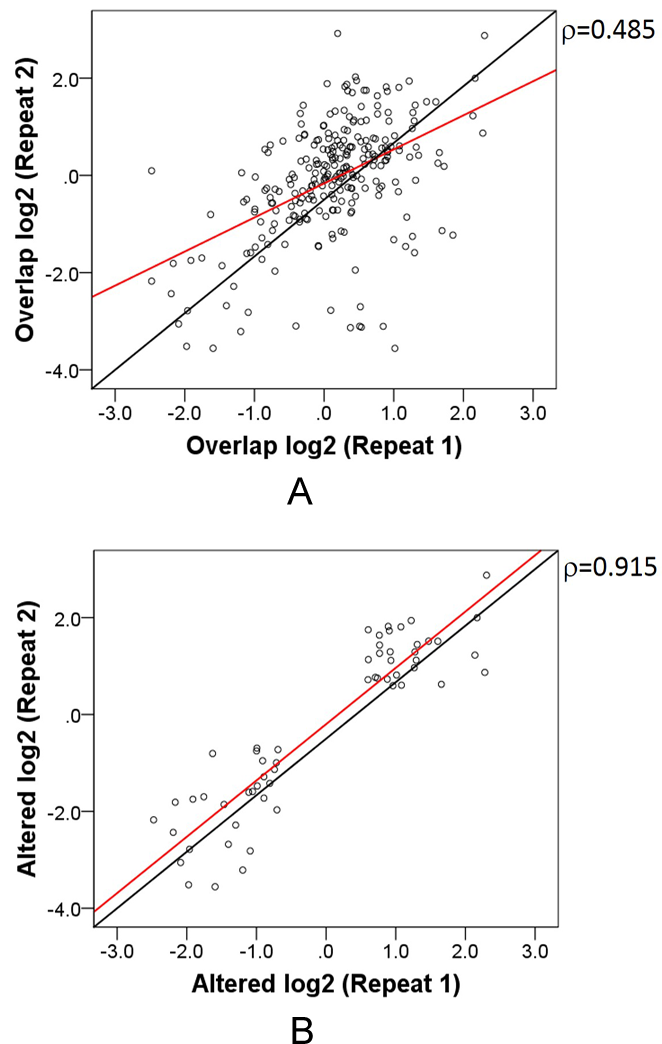

Supplement: Figure S1 — Correlation analysis of overlapped proteins and differential proteins (log2 ratio) between two biological replicates. (A,B) the correlation of commonly quantitative and altered proteins from two biological replicates, respectively. [file Image_1.TIF]

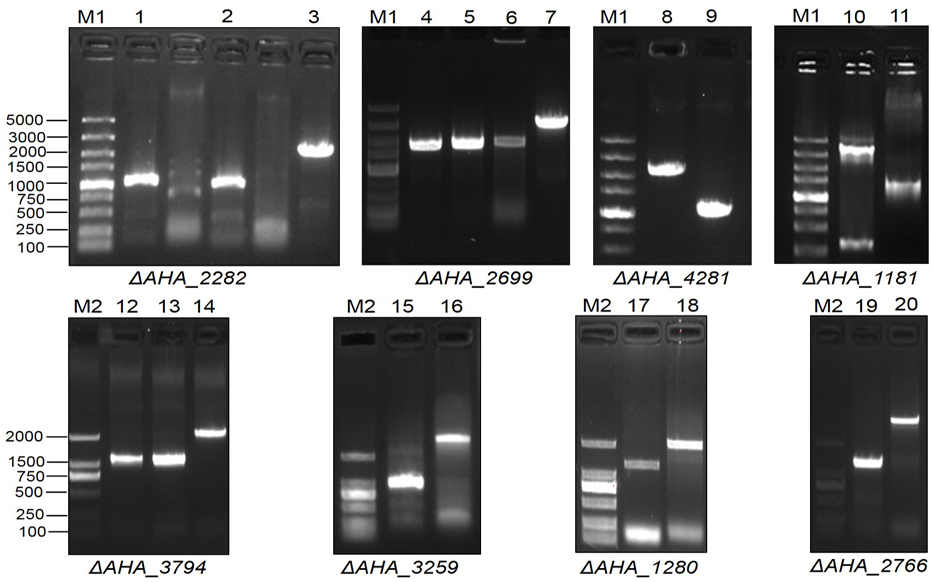

Supplement: Figure S2 — Validation of the knockout mutants by colony PCR amiplified with the primer pairs P7 and P8. The sizes of eight genes are 2424 bp (AHA_1181), 1002 bp (AHA_1280), 1638 bp (AHA_2282), 1344 bp (AHA_2699), 1992 bp (AHA_2766), 1191 bp (AHA_3259), 570 bp (AHA_3794), and 1647 bp (AHA_4281), respectively. Lanes M1 and M2, the DL 5,000 and DL 2,000 DNA marker; lanes 3, 7, 8, 10, 14, 16, 18, and 20, the PCR products of wild-type strains amplified with the primers P7 and P8 as a positive control, and their expected sizes of fragments were 2966 bp (lane 3), 3133 bp (lane 7), 2847 bp (lane 8), 3594 bp (lane 10), 1810 bp (lane 14), 2396 bp (lane 16), 2166 bp (lane 18), and 3165 bp (lane 20); lanes 1, 2, 4, 5, 6, 9, 11, 12, 13, 15, 17, and 19, the possible colonies of knockout mutants pick out for colony PCR verification with primers P7 and P8; in addition to lanes 4-6 (1789 bp), the expected sizes of remaining ones were 1328 bp (lane 1-2), 1200 bp (lane 9), 1170 bp (lane 11), 1240 bp (lane 12-13), 1205 bp (lane 15), 1164 bp (lane 17), and 1173 bp (lane 19), which means the right results that these genes have been deleted in A. hydrophila. [file Image_2.TIF]

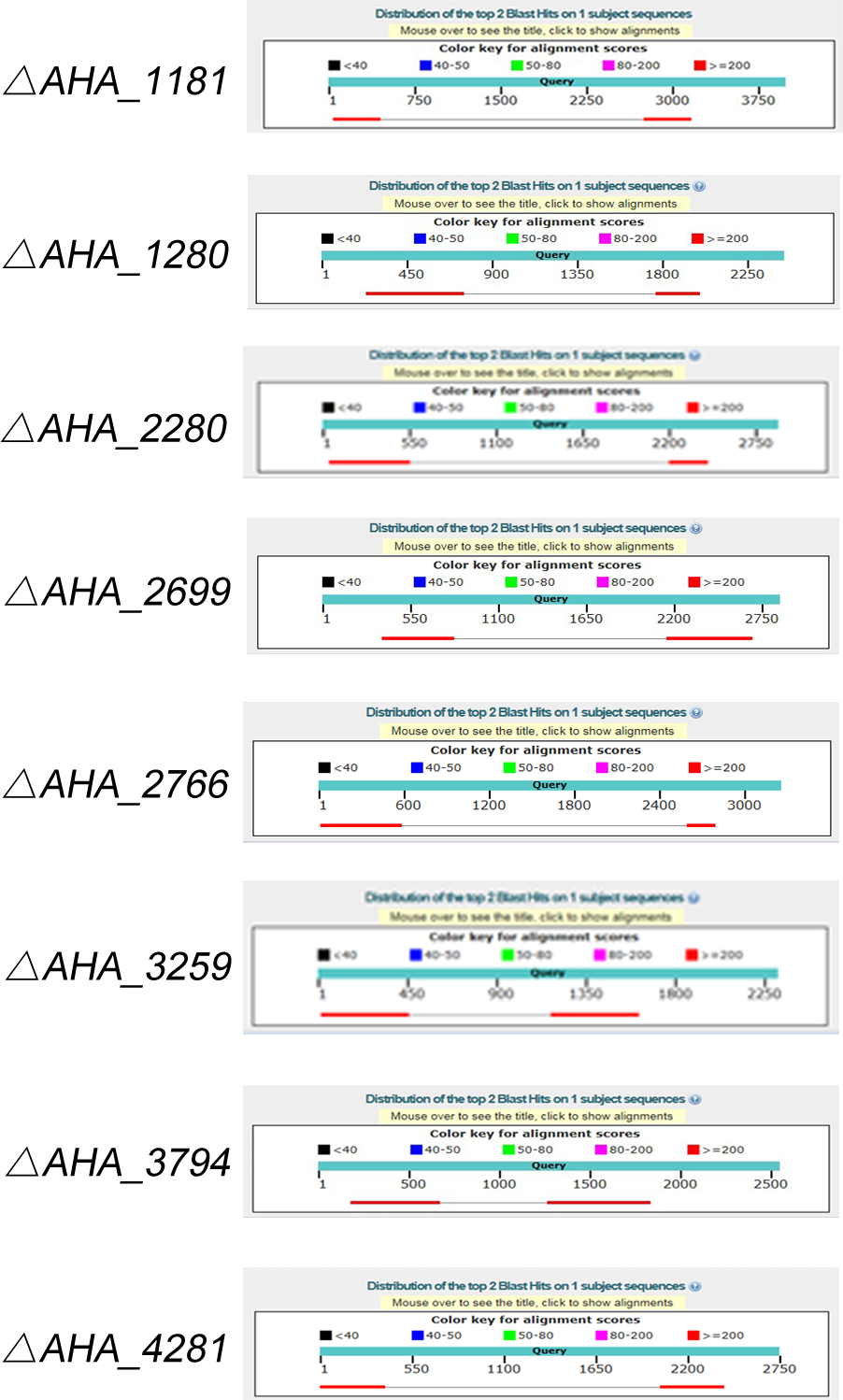

Supplement: Figure S3 — Comparisons from sequencing results of the genetic deletion mutants by BLAST online software. The possible mutants were sequenced with P7 and P8 primers and compared to their genes in A. hydrophila used on online software BLAST. [file Image_3.TIF]

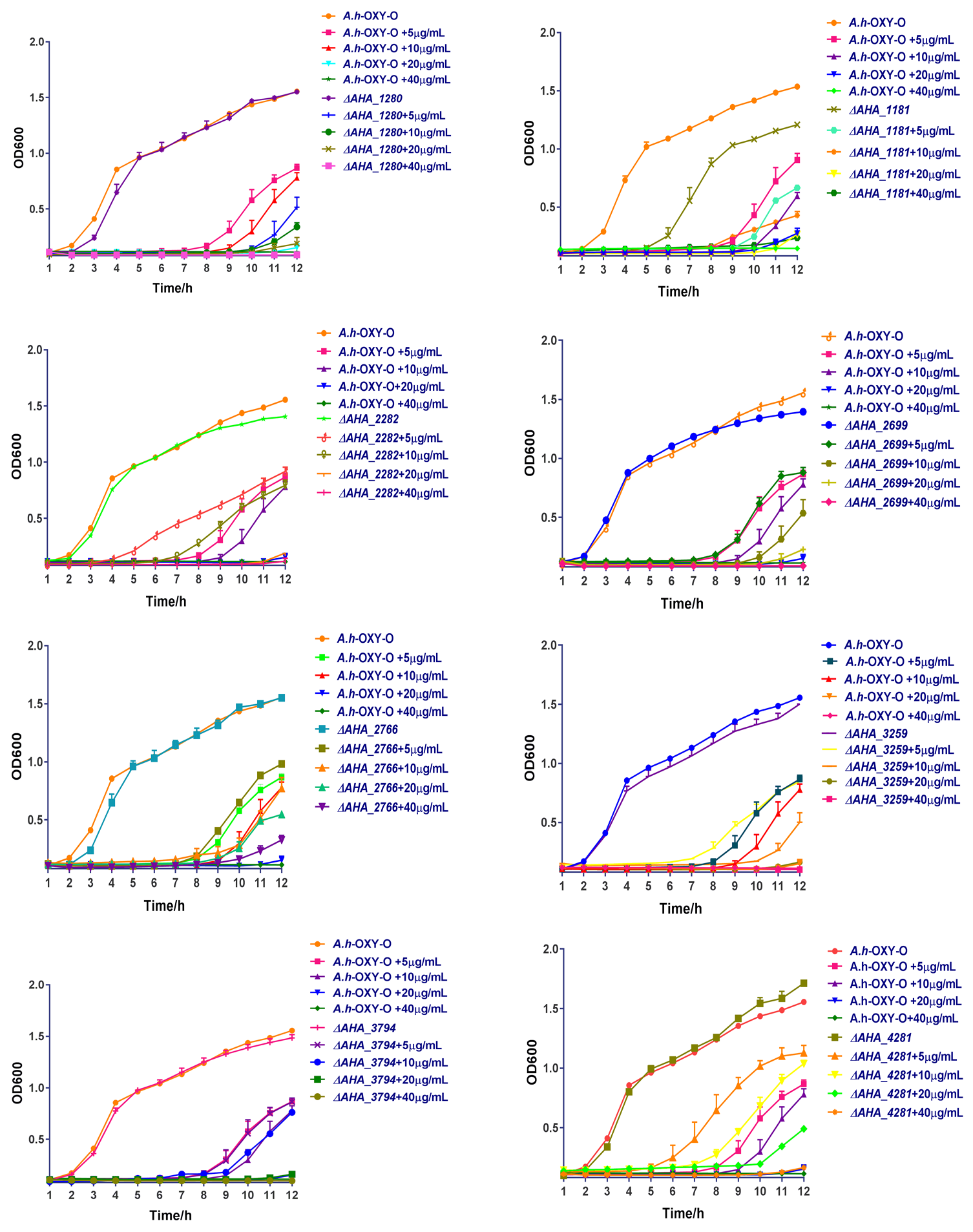

Supplement: Figure S4 — Growth curves of the mutants and A.h- OXY-O under serial concentrations of OXY stress. The growth curves of wile-type strain and mutants were measured for 12 h in different concentrations of 0, 5, 10, 20, and 40 μg/mL OXY. [file Image_4.TIF]

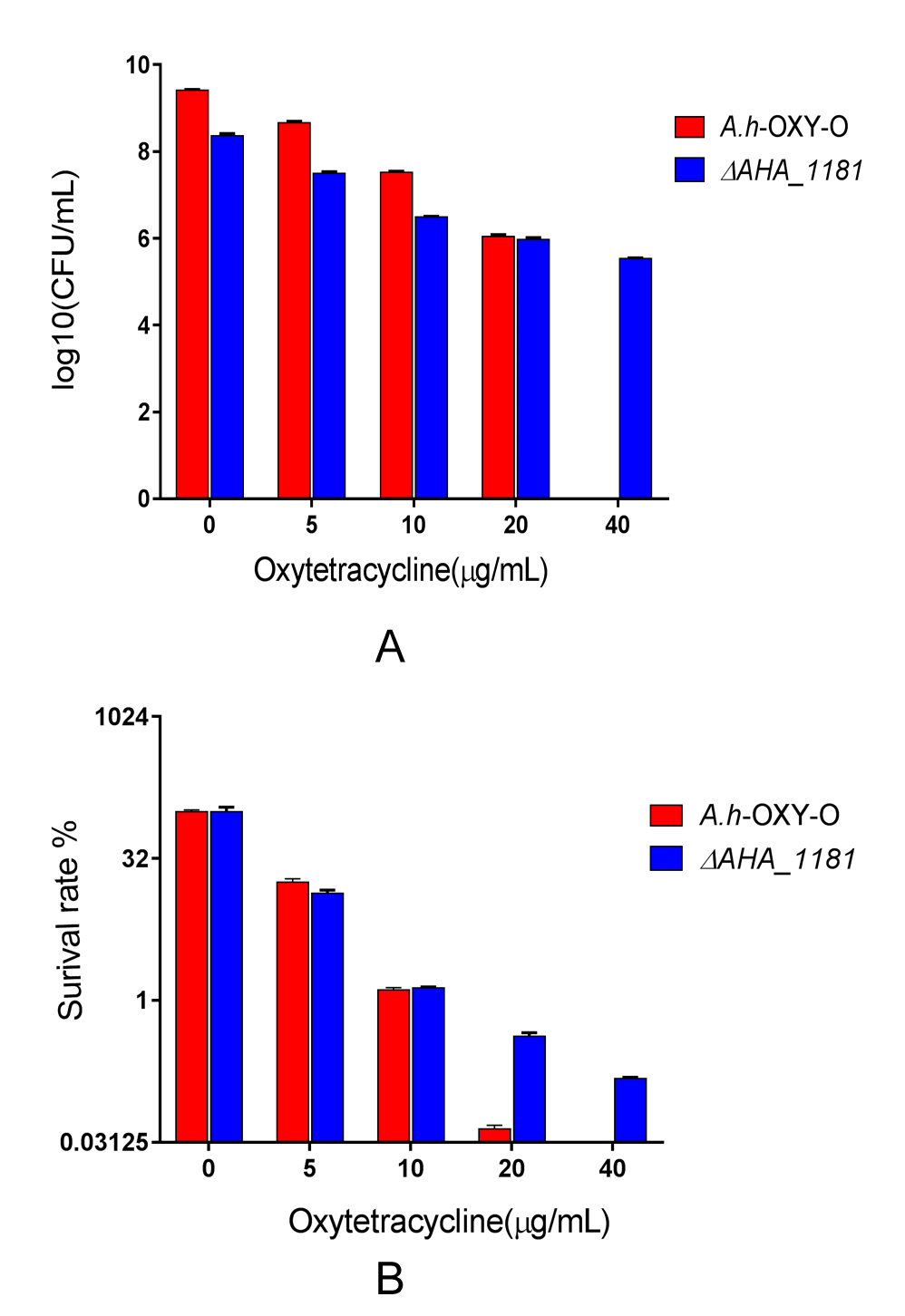

Supplement: Figure S5 — Survival capabilities of A.h- OXY-O and ΔAHA_1181 treated with a series of OXY concentrations (0, 5, 10, 20, and 40 μg/mL) for 12 h and observed by colony counting. (A) the CFU of them under each concentration of OXY treatment (log10 scale on the y-axis). (B) the survival ratio of wild type and ΔAHA_1181 mutant when treated with serial dilutions of OXY concentrations for 12 h. [file Image_5.TIFF]

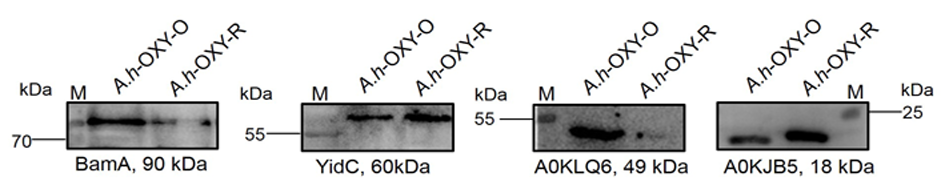

Supplement: Figure S6 — Western blotting analysis for differential outer membrane proteins contained the prestained protein ladder. The prestrained protein marker was used to detect the molecular weights of proteins, and the expected size of BamA. YidC, A0KLQ6, and A0KJB5 was 90 kDa, 60 kDa, 49 kDa, and 18 kDa, respectively. [file Image_6.TIF]

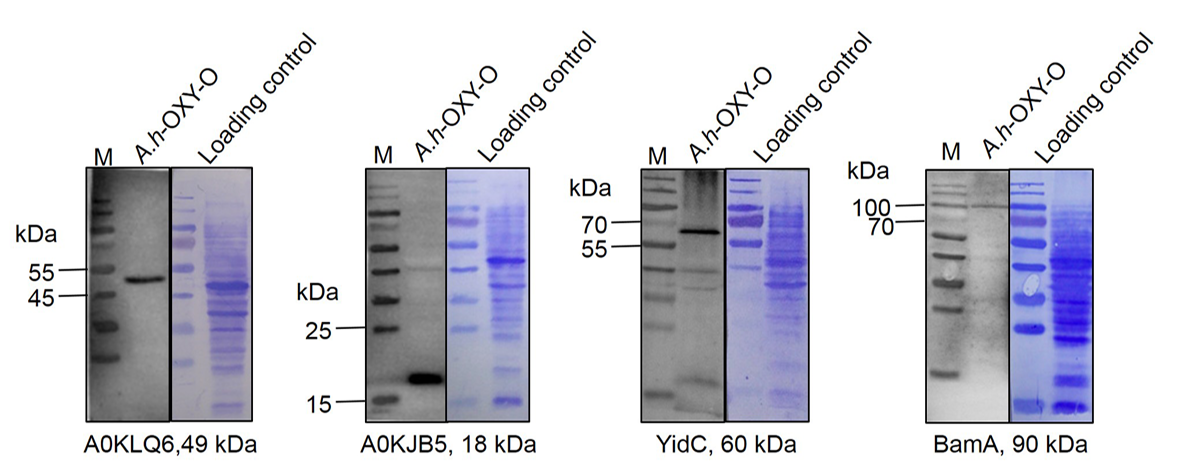

Supplement: Figure S7 — The validation of the specificity of antibodies by Western blotting. Western blotting was used to detect the specificity of these polyclonal antibodies in A.h-OXY-O. Coomassie staining was used as the loading control (on the right). The expected size of A0KLQ6, A0KJB5, YidC, and BamA was 49 kDa, 18 kDa, 60 kDa, and 90 kDa, respectively. [file Image_7.TIF]

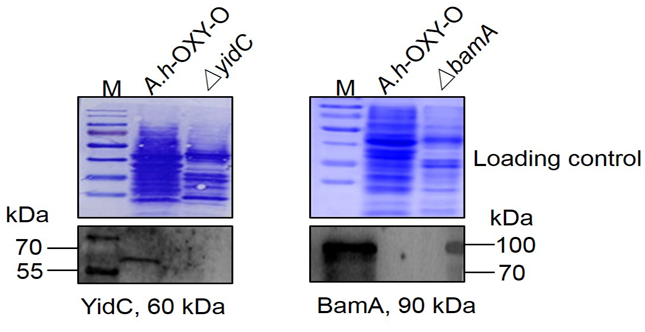

Supplement: Figure S8 — The specificity of anti-YidC and BamA was further validated in knock-out strains by western blotting. The specificity of two antibodies were examined in ΔAHA_4281 (yidC) and ΔAHA_1181 (bamA), and coomassie staining was used as the loading control. [file Image_8.TIF]
